# Giant Cell Tumor of the Tendon Sheath: Experience With 65 Cases

**Published:** 2012-11-12

**Authors:** Erin L. Adams, Eric M. Yoder, Morton L. Kasdan

**Affiliations:** ^a^University of Louisville School of Medicine, Louisville, Ky; ^b^Division of Plastic and Reconstructive Surgery, University of Louisville, Louisville, Ky

## Abstract

**Objective:** No consensus exists on the etiology, prognostic factors, or recurrence rate of giant cell tumors of the tendon sheath. This article presents a series of 65 cases supplemented by a literature review that examines the epidemiology, presentation, gross and microscopic characteristics, and recurrence rate of giant cell tumor of the tendon sheath.

**Methods:** The authors completed a retrospective review of one surgeon's practice from 1976 to 2001, evaluating 65 cases of giant cell tumor of the tendon sheath. The authors conducted a literature search and compared the case series with historical data.

**Results:** The tumor most commonly presented as a firm, nontender mass in the dominant hand. Our cases showed a slight female predominance of 54%, compared with the literature average of 64%. A pseudocapsule was present in 51% of cases. Overall recurrence rate was 10%. No association was noted between recurrence and pseudocapsule presence, rheumatoid arthritis, or osteoarthritis. Satellite lesions at the first excision were noted in 80% of recurrent cases; however, satellite lesions were not a risk factor for recurrence per se.

**Conclusions:** Our study shows similar findings to the literature, with the notable addition of satellite lesions in recurrent tumors. Marginal excision is the treatment of choice, but may be complicated when the tumor is attached to vital structures. Therefore, an appropriate balance between resection of tumor and maintenance of function must be achieved due to the possibility of recurrence.

Giant cell tumor of the tendon sheath (GCTTS) is a benign tumor, presenting as the second most common mass of the hand after ganglion cysts.^1^ It was first described by Chassaignac in 1852 as fibrous xanthoma^2^ and has since been referred to by multiple names, including localized nodular tenosynovitis, pigmented villonodular proliferative synovitis, sclerosing hemangioma, benign synovioma, proliferative synovitis, xanthoma, xanthogranuloma, xanthosarcoma, myeloid endothelioma, fibrohemosideric sarcoma, giant cell fibrohemangioma, pigmented villonodular tenosynovitis, fibroma, myeloma, myeloxanthoma, and fibrous histiocytoma.^1^^,^^3^^-^5 The large range of nomenclature indicates disagreement as to the etiology of giant cell tumors. The prevailing divergence is between a neoplastic^1^^-^3^,^^6^^-^9 and inflammatory origin^10^^-^12 of the tumor, with multiple studies presenting evidence for each.

Despite the undetermined etiology, the clinical presentation, diagnosis, and surgical treatment of GCTTS are described. The tumor is most commonly diagnosed in the fourth and fifth decades of life (range, 4-82 years),^13^^,^^14^ with women affected more commonly than men (64.3% women).^2^^,^^3^^,^^15^ Although GCTTS most commonly presents in a digit of the hand, it may also present in the palm,^1^^,^^16^ wrist,^2^^,^^17^ foot,^7^^,^^13^^,^^18^ knee,^7^^,^^13^^,^^18^ ankle,^13^ elbow,^13^ or hip.^2^ Grossly, GCTTS is a multilobular and generally well-circumscribed tumor. It may be partially or completely encapsulated and may have extensions and/or satellite lesions connected by as little as a few strands of fibrous tissue.^6^^,^^15^ Coloration varies from gray to yellow-orange with some brownish areas, depending on the amounts of hemosiderin, collagen, and histiocytes present in the tumor.^15^ Histologically, giant cell tumor is composed of 4 main cell types, namely the principal synovial cell, multinucleated giant cell, foam cell, and histiocyte-like cell.^18^ These cells are contained within a fibrous collagenous stroma, form synovial-lined spaces, and are often surrounded by a thin, fibrous capsule.^6^^,^^13^^,^^18^

Giant cell tumor of the tendon sheath is most commonly found in the distal interphalangeal (DIP) joint^2^^,^^15^^,^^19^ and the proximal phalanx.^2^^,^^4^^,^^20^^-^22 Some authors describe an association with rheumatoid arthritis,^15^ while others describe an association with osteoarthritis;^2^^,^^4^ however, these findings are not replicated across large numbers of studies.

## Diagnosis

Giant cell tumor of the tendon sheath most commonly presents as a firm, nontender, nonfluctuant nodule in a digit of the hand. The most common occurrence is in the index finger (26%), followed by the long finger, thumb, ring finger, and small finger, respectively (Table 1). The tumor is predominantly palmar,^1^^,^^4^^,^^16^^,^^19^^,^^20^^,^^23^ although Jones et al^2^ and Ushijima et al^13^ reported dorsal tumors to be more prevalent. Because of the slow-growing nature of the tumor, patients present an average of 6 months to 2.5 years after the initial onset of symptoms.^6^^,^^20^

Diagnosis of GCTTS is largely made by clinical examination; however, giant cell tumor may be misdiagnosed or left without definitive diagnosis until intraoperative findings are available. For example, Monaghan reports a provisional diagnosis of GCTTS in only 4.2% of 71 affected patients, with the remainder of tumors diagnosed as ganglion or epidermoid cyst.^18^ In less extreme cases, 41.5%,^7^ 87.5%,^23^ and 92%^16^ of tumors were diagnosed as GCTTS preoperatively.

No characteristic radiographic appearance is seen with GCTTS.^8^ Roentgenographic findings indicate a soft tissue mass in the majority of cases; however, Reilly et al^4^ reported normal findings in 32% of patients. Ultrasonography shows a solid, homogeneous, hypoechoic mass generally in relation to the flexor tendons of the fingers, with increased vascularity on Doppler studies.^19^ Magnetic resonance imaging reveals decreased signal intensity on T1- and T2-weighted images.^8^ A preoperative pathological diagnosis can be made using fine-needle aspiration biopsy.^24^ Although radiography cannot be used to make a definitive diagnosis, it is useful in showing bony erosion and is widely available. Magnetic resonance imaging is the most definitive imaging study;^24^ however, it is generally not obtained due to the ubiquity and cost-effectiveness of radiography and ultrasound.

## Treatment

Treatment for giant cell tumor is local excision. Care must be taken to preserve the flexor tendons, extensor tendons, digital arteries, and nerves if possible. Because of the usual presence of a pseudocapsule, the tumor can often be removed en bloc. All surrounding tissues should be examined for satellite lesions, and such lesions and connections to these lesions should be excised. Rather than opening the entire site, satellite lesions can often be removed using a teasing technique,^15^ which utilizes gentle, slow dissection. If erosion of the bone has occurred, curettage to remove the cortical shell is advised.^3^ Flexor and extensor tendons invaded by the tumor should be repaired.

Recurrence is a major concern in GCTTS, with rates of up to 44% being reported.^6^ In the case of recurrence, marginal excision of the tumor should be repeated. Functionality of the involved digit should be considered and may result in the decision to amputate for large tumors that interfere with function.

Radiotherapy has been indicated as an adjuvant therapy for the prevention of recurrence. Garg and Kotwal^16^ reported dramatic reductions in recurrence rates, with total recurrence rates of 0% and 4%, respectively, with the use of radiotherapy after surgery.^23^

## METHODS

The authors examined a series of 65 cases of GCTTS from one surgeon's (M.L.K.) practice between 1976 and 2001. Parameters including, but not limited to, presentation, age, size, location, recurrence, and pseudocapsule were compiled in a database using Microsoft Excel. Data analysis was used to analyze various factors and associations.

## RESULTS

A total of 27 male and 35 female patients presented at a mean age of 49 years (range, 8-80). Two patients had multiple tumors upon presentation. Patients presented an average of 27 months after initial onset of symptoms (range, 2 weeks-16 years). Patients were followed for an average of 5.7 years (range, 1 week-17.5 years).

**Figure 1:**
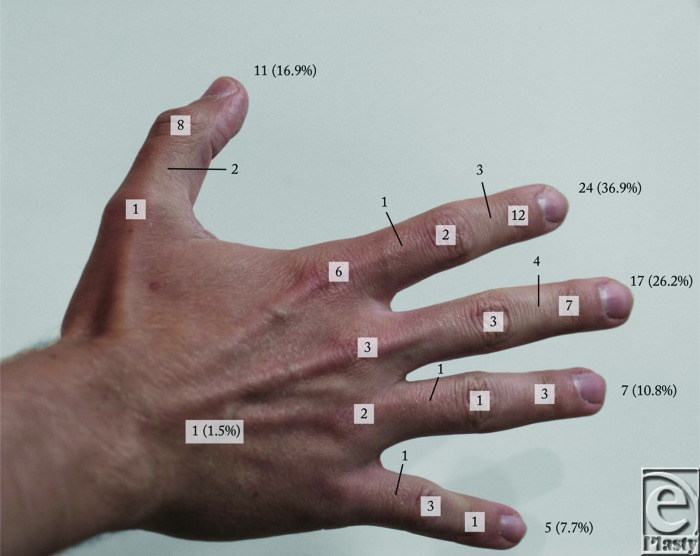
Location of giant cell tumor of the tendon sheath in current study. Boxes and lines indicate the number of tumors at each joint and phalanx, respectively. At the tip of each respective digit, the total number is shown with percent of total indicated in parentheses.

Patients most often presented with a nontender, slow-growing nodule with no history of trauma and no associated numbness or tingling (Table 2). Tenderness was noted in 31% of patients, trauma was reported in 14%, and associated numbness or tingling was reported in 9% of cases. The affected hand was dominant in 67% of cases and was the right hand in 65% of cases. The tumor was most often located on the palmar aspect of the hand (49%), followed by the dorsal aspect (35%), ulnar aspect (11%), and radial aspect (5%). The most common digital location was the index finger (37%). The most common area in which the tumor was located was the DIP joint (35%). Looking together at the digital and joint/phalanx locations, the tumor was most frequently located at the DIP joint of the index finger (Fig 1). No association was noted between GCTTS and rheumatoid arthritis or osteoarthritis.

All procedures were performed under either 3.6x or 4.5x magnification. Tenolysis was required for complete resection in 54% of cases. The mean diameter of the tumor measured 1.5 cm (range, 0.5-3.5). A description of the tumor as encapsulated or well circumscribed was given in 51% of cases. Satellite lesions were noted in 11 cases (17%). Bony erosion was evident in 18 cases (28%), but intraosseous invasion never occurred. No surgical complications were noted in any of the cases.

Of the 50 patients followed for at least 1 year, 5 patients experienced recurrence, with an average of 4.2 years (range, 2.5-9.2) between initial excision and recurrence. One patient experienced 2 recurrences, with the recurrences occurring after 3.5 and 2 years, respectively. This patient was followed for 7 years subsequent to the removal of the second recurrent tumor and experienced no further recurrence.

In one case, the surgeon chose to amputate the affected digit because of the extensive nature of the tumor, which impaired hand function and increased the likelihood of recurrence.

### Case example

**Figure 2:**
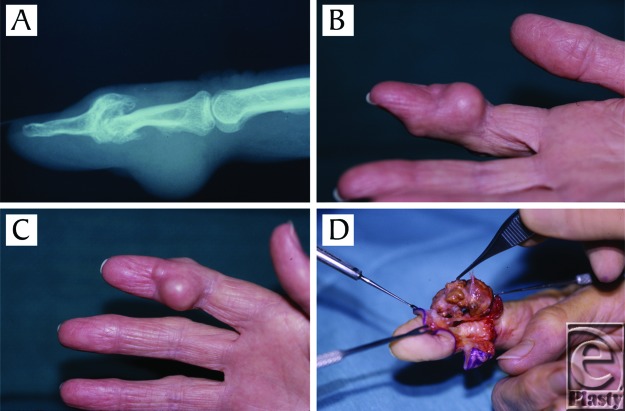
A 64-year-old woman with giant cell tumor of the tendon sheath (GCTTS) on the palmar aspect of the middle phalanx of the right index finger. (*a*) X-ray showing bony erosion. (*b*)-(*c*) Lesion upon presentation. (*d*) Intraoperative view showing typical multilobulated appearance of GCTTS.

A 64-year-old woman presented with an asymptomatic tumor that had been present and increasing in size for the past 6 years (Fig 2). The tumor presented primarily on the palmar aspect of the middle phalanx of the right index finger. The patient had a history of degenerative joint disease. Tumor size and bony erosion were visible on x-ray films. Intraoperatively, the multilobulated, gray-brown to yellow appearance of GCTTS was well visualized. Meticulous dissection allowed preservation of most structures, but the dorsal digital nerves and the digital artery were sacrificed because of extensive involvement. Tenolysis was required for adequate excision. The tumor was 2.5 cm in diameter and satellite lesions were noted. No recurrence was present after 1 year of follow-up.

## DISCUSSION

The diagnosis, treatment, and recurrence of GCTTS are widely debated topics. Many authors have described varying etiologies and prognostic factors for recurrence. This complicates the management of patients who may have GCTTS. The variability of a preoperative diagnosis of GCTTS between studies indicates a discrepancy in diagnostic techniques and/or knowledge of tumor presentation. Recurrence rates have ranged from 0%^18^^,^^23^ to 44%,^6^ which may be attributed to the balance of minimal excision of a benign process with maintenance of function in a highly important location. Adjuvant therapies such as phenol treatment^5^ and radiation therapy^16^^,^^23^ have been described to lower recurrence rates; however, neither of these methods were used in the current study. Prognostic factors such as tumor location,^4^ pseudocapsule presence,^16^^,^^25^ bony erosion,^4^^,^^9^ tissue involvement,^22^ and nm23 gene expression^9^ have all been described. This wide range of findings suggests that there is no consensus on etiology, prognostic factor, or optimal treatment for GCTTS.

As with the removal of any lesion, the goal of the surgeon in dealing with GCTTS is to excise the tumor with minimal disruption to adjacent structures. However, adequate excision may be compromised in an effort to fully preserve anatomical structures. Because recurrence is thought to be due to incomplete excision of the tumor or satellite lesions, a balance must be maintained between salvaging tissue and adequately removing tumor margins. Increased recurrence has been associated with distal joint location of the tumor^4^ and direct involvement of the tumor with the extensor tendon, flexor tendon, or joint capsule.^22^ This is likely due to more difficult excision of such tumors. In light of these findings, it has been suggested that the surgeon be more aggressive in removing tumors that are directly involved with these structures.^22^ The reported recurrence rates of GCTTS appear to be decreasing over time but remain variable between studies. This variation may be due to publication bias, but could also be ascribed to different surgical techniques, with inadequate tumor removal resulting in an increased risk of recurrence. To minimize recurrence, complete marginal excision should be performed.

This study was limited due to the retrospective nature of the case series, which depends on the accuracy and detail of the medical record and is uncontrolled. Only 1 surgeon's experiences were included in this study. In addition, this surgeon compiled more than 200 cases of GCTTS during his practice in the 1970s to 2000s, but only 64 charts were located. This factor could have led to a skewed sample that may have affected the statistics in this article. Future research could entail a prospective, controlled study, which might look at various topics such as the recurrence effect predicted by pseudocapsule presence, bony erosion, presence of satellite nodes, or tumor location.

## Figures and Tables

**Table 1:** Anatomic location of giant cell tumor of the tendon sheath in the literature

	Thumb	Index finger	Long finger	Ring finger	Small finger	Palm, wrist, or dorsum of hand	Total
Al-Qattan^25^	1	15	15	4	8	0	43
Darwish and Haddad^24^	19	10	5	5	7	5	51
Garg and Kotwal^16^	14	28	18	36	6	4	106
Jones et al^2^	15	24	20	12	20	0	91
Kotwal et al^23^	4	16	10	12	4	2	48
Middleton et al^19^	4	3	1	3	1	0	12
Phalen et al^1^	7	12	18	11	7	2	57
Reilly et al^4^	21	27	21	21	17	0	107
Rodrigues et al^26^	5	7	2	2	1	1	18
Uriburu and Levy^21^	4	3	1	3	4	0	15
Ushijima et al^13^	37	38	37	23	23	6	164
Wright^6^	12	10	11	5	6	13	57
Current study	11	24	17	7	5	1	65
Total	154	217	176	144	109	34	834
Percentage	18.5%	26.0%	21.1%	17.3%	13.1%	4.1%	

**Table 2:** Demographic data of current study

Parameter	%
Associated numbness/tingling	9.2
Associated tenderness	30.8
Associated trauma	13.8
Bony erosion	27.7
Degenerative joint disease	10.8
Dominant hand	67.2
Intraosseous invasion	0.0
Male	46.2
Pseudocapsule	50.8
Rheumatoid arthritis	3.1
Recurrence	10.0
Right hand	64.6
Satellite lesion(s)	16.9
Tenolysis performed	53.8
